# Serum advanced glycation end-products as a biomarker of cumulative glycaemic burden and complications in type 2 diabetes: a pilot study

**DOI:** 10.1080/07853890.2026.2671517

**Published:** 2026-05-20

**Authors:** Xiuqin Lin, Danping Lv, Weifeng Meng, Panpan Wang, Xinyuan Zhang

**Affiliations:** ^a^ Department of Laboratory, Shaoxing Seventh People’s Hospital, Shaoxing, Zhejiang, China; ^b^ Department of Geriatric Psychiatry, Shaoxing Seventh People’s Hospital, Shaoxing, Zhejiang, China

**Keywords:** Advanced glycation end products, type 2 diabetes mellitus, complications, prediabetes, biomarkers

## Abstract

**Background:**

Advanced glycation end products (AGEs) link chronic hyperglycaemia to diabetic tissue damage. Serum AGE concentrations across the metabolic disease spectrum, from prediabetes through complicated diabetes, remain incompletely characterized, including the contribution of isolated dyslipidaemia.

**Objective:**

To quantify serum AGEs across metabolic states and evaluate their biomarker potential for disease staging and complication risk stratification.

**Methods:**

Cross-sectional study of 226 adults in five groups: healthy controls, isolated hyperlipidaemia, impaired glucose tolerance, uncomplicated type 2 diabetes, and complicated type 2 diabetes. Serum AGEs were measured by ELISA. Group differences were assessed by Kruskal–Wallis tests, logistic regression, and receiver operating characteristic analysis.

**Results:**

Serum AGEs increased progressively across groups (Kruskal–Wallis *H* = 79.95, *p* < .001). Compared with healthy controls (median 5.99 AU), AGEs showed a trend in impaired glucose tolerance (7.22 AU; Cohen’s *d* = 0.425; uncorrected *p* = .037, corrected *p* = .094), significant increases in uncomplicated diabetes (9.81 AU; *d* = 1.027, *p* < .001) and complicated diabetes (13.35 AU; *d* = 1.540, *p* < .001), with no elevation in isolated hyperlipidaemia (5.86 AU; *p* = .987). AGEs discriminated uncomplicated from complicated diabetes (*d* = 0.802, *p* < .001) where HbA1c (*d* = 0.022, *p* = .706) and fasting glucose (*d* = 0.023, *p* = .908) did not. ROC analysis showed excellent discrimination of complicated diabetes from controls (AUC = 0.908, 95% CI [0.834–0.970]). AGEs remained an independent predictor of complicated diabetes (adjusted OR = 4.92, *p* = .002) and correlated with complication burden (ρ = 0.332, *p* = .016).

**Conclusion:**

Serum AGEs rise progressively from prediabetes to complicated diabetes, driven by hyperglycaemia. Their discriminative capacity where conventional glycaemic markers plateau supports AGEs as a complementary biomarker for complication risk stratification, pending validation.

## Graphical abstract

**Figure:**
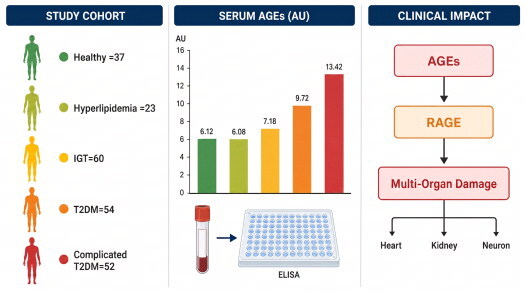

Highlight Box

**What is already known about this topic?**

- Advanced glycation end products (AGEs) are key mediators linking hyperglycaemia to diabetic tissue damage.
- AGE accumulation is associated with chronic complications in established type 2 diabetes mellitus (T2DM).
- Conventional markers like HbA1c reflect medium-term glycaemic control but may not capture cumulative biomolecular damage.

**What does this research add?**

- Serum AGEs showed progressive accumulation across the metabolic disease continuum, from normoglycemia to complicated type 2 diabetes, with a trend toward elevation already apparent at the prediabetic stage. AGE elevation appeared specific to glycemic dysregulation, as isolated hyperlipidemia was not associated with increased AGE levels. In addition, AGEs retained the ability to distinguish uncomplicated from complicated diabetes where conventional glycemic markers showed limited discriminative capacity, supporting their potential role as complementary indicators of cumulative metabolic injury beyond HbA1c alone.

**What is the clinical significance?**

- Positions AGE measurement as a promising tool for early risk stratification during the prediabetic window.
- Provides a quantitative index of cumulative glycaemic burden beyond conventional markers.
- Offers a potential biomarker to identify patients at high risk for multi-organ complications, enabling earlier intervention.

## Introduction

Type 2 diabetes mellitus (T2DM) accounts for over 90% of diabetes cases globally and develops along a continuum from normal glucose tolerance through impaired glucose tolerance (IGT) to overt disease [1]. Individuals with impaired glucose tolerance (IGT) are at a substantially increased risk of progressing to type 2 diabetes mellitus over time, a process largely driven by worsening insulin resistance and progressive β-cell dysfunction [2]. Current estimates indicate 537 million adults have diabetes worldwide, projected to reach 783 million by 2045 [3]. Beyond glycaemic dysregulation, T2DM precipitates severe complications: cardiovascular risk increases two to four-fold, nephropathy underlies one-third of end-stage renal disease, retinopathy causes most working-age blindness, and neuropathy accounts for over half of non-traumatic amputations [4,5].

Conventional glycaemic markers such as HbA1c reflect glucose exposure over weeks to months and may incompletely capture cumulative metabolic damage driving long-term complications. Advanced glycation end products (AGEs) [6] offer a complementary perspective by integrating prolonged glycaemic and oxidative stress. AGEs form through non-enzymatic reactions between reducing sugars and amino groups of macromolecules, a process accelerated under hyperglycaemic conditions [7,8].

AGEs promote tissue injury through two mechanisms: direct modification of extracellular matrix proteins impairing vascular integrity, and receptor-mediated activation of inflammatory cascades *via* RAGE signalling [9]. Clinical evidence links AGE levels to nephropathy, retinopathy, and cardiovascular disease severity [10]. More recent studies, utilizing advanced analytical methods, have demonstrated that AGE-derived metrics, such as the AGE z-score, strongly correlate with the presence of both macrovascular and microvascular complications, suggesting their utility as a direct marker of disease severity [11]. Recent data further implicate the AGE-RAGE axis in β-cell dysfunction, potentially accelerating diabetes progression itself [12].

Despite growing recognition of AGE pathogenicity, most studies have examined isolated patient populations rather than the complete metabolic disease trajectory. This gap limits understanding of how AGE accumulation evolves from health through prediabetes to complicated diabetes, and whether AGEs can reliably discriminate between disease stages. The continued emergence of evidence linking AGEs to the pathogenesis and severity of T2DM complications underscores the need for a comprehensive assessment of AGE dynamics across the full metabolic spectrum [13]. To address this, we measured serum AGE concentrations across five clinically defined groups spanning the metabolic continuum: healthy controls, patients with hyperlipidaemia, individuals with IGT, patients with uncomplicated T2DM, and patients with complicated T2DM. We further analysed correlations between AGEs and conventional clinical parameters. This comprehensive approach aimed to delineate AGE dynamics throughout T2DM evolution and assess their potential utility for risk stratification and early intervention.

## Materials and methods

### Study population and design

Our pilot study enrolled 226 participants attending Shaoxing Seventh People’s Hospital between January 2024 and June 2025. Participants were stratified into five groups: healthy controls (HC, *n* = 37), patients with hyperlipidaemia (HL, *n* = 23), individuals with impaired glucose tolerance (IGT, *n* = 60), patients with uncomplicated type 2 diabetes mellitus (T2DM, *n* = 54), and patients with T2DM complicated by end-organ damage (T2DM + C, *n* = 52). Within the T2DM + C group, 31 patients had a single complication, 16 had two complications, and 5 had three or more complications. Groups were not matched 1:1; sample sizes reflect consecutive enrolment of eligible participants attending endocrinology and health screening clinics. Age and sex distributions differed across groups, with older age and higher male predominance in advanced disease stages (Table S1). These demographic imbalances are acknowledged as a limitation and were addressed through multivariable adjustment in secondary analyses.

### Inclusion criteria

Participants were eligible if they met the following criteria: age between 34 and 75 years; T2DM diagnosis conforming to the 1999 World Health Organization diagnostic and classification standards; provision of written informed consent following institutional ethics committee approval; and absence of high-fat dietary habits or unusual dietary preferences as determined by pre-enrolment dietary questionnaire, ensuring comparable dietary patterns across groups.

### Exclusion criteria

Participants were excluded if they had: (1) type 1 diabetes mellitus or secondary diabetes (e.g. steroid-induced, pancreatic disease); (2) acute infection, systemic inflammatory disease, or malignancy within the preceding 3 months; (3) severe hepatic impairment (ALT or AST >3× upper limit of normal) or severe renal impairment (estimated glomerular filtration rate [eGFR] < 30 mL/min/1.73m^2^); (4) current pregnancy or lactation; (5) history of bariatric surgery or other major gastrointestinal surgery affecting nutrient absorption; (6) use of exogenous AGE-modifying therapies (e.g. alagebrium chloride) or participation in clinical trials within 6 months; or (7) inability to provide informed consent. Participants with unusual dietary habits (e.g. strict vegan, very high-protein, or ketogenic diets) or high exogenous AGE intake as determined by a pre-enrolment dietary screening questionnaire were also excluded to minimize dietary confounding.

### Sample collection and laboratory measurements

All participants fasted for 12 h prior to sample collection. Venous blood was drawn the following morning: 5 mL into serum separator tubes for biochemical analysis and 2 mL into EDTA-anticoagulated tubes for HbA1c measurement.

Glycated haemoglobin was quantified using a Pumen H9 HbA1c analyser after thorough mixing of anticoagulated whole blood. For AGE determination, serum separator tubes were maintained at room temperature for 1 hour, then centrifuged at 2000 × g for 10 min at 4 °C. Serum was collected and AGE concentrations were measured by enzyme-linked immunosorbent assay (ELISA) using Yousibio reagent kits on a Molecular Devices Cmax Plus microplate reader. Renal function (creatinine, eGFR calculated by the CKD-EPI equation), liver function (alanine aminotransferase [ALT], aspartate aminotransferase [AST], gamma-glutamyl transferase [GGT]), and lipid profiles (total cholesterol, LDL-cholesterol, HDL-cholesterol, triglycerides) were analysed on an Abbott ARCHITECT c16000 automated biochemistry analyser following centrifugation at 3500 rpm for 5 min.

An important methodological consideration is that the polyclonal antibody used in this ELISA recognizes immunoreactive AGE species and lacks molecular specificity for individual AGE structures such as Nε-(carboxymethyl)lysine (CML), Nε-(carboxyethyl)lysine (CEL), or methylglyoxal-derived hydroimidazolone (MG-H1). The assay quantifies total immunoreactive AGE content rather than individual molecular adducts, which should be considered when interpreting results

## Statistical analysis

Continuous variables are expressed as mean ± standard deviation (SD) and median (interquartile range [IQR]) for non-normally distributed data, while categorical variables are presented as frequencies and percentages. Distributional normality was evaluated using the Shapiro-Wilk test. Given the non-normal distribution of AGE data, non-parametric statistical methods were employed throughout. Although non-parametric methods were used for all inferential statistics, means and standard deviations are additionally provided to facilitate comparison with prior literature that predominantly reports parametric descriptors.

Between-group comparisons were conducted using the Kruskal–Wallis H test. Post-hoc pairwise comparisons were performed using Dunn’s test with Bonferroni correction to adjust for multiple testing. Correlations between AGE levels and clinical parameters were assessed using Spearman’s rank correlation coefficient (ρ). Partial Spearman correlations controlling for age were computed to assess robustness to age-related confounding.

Receiver operating characteristic (ROC) curves were constructed to evaluate the diagnostic performance of AGEs for discriminating between study groups. Area under the curve (AUC) values with corresponding 95% confidence intervals were calculated using 2000 bootstrap resamples. Optimal cutoff values maximizing diagnostic accuracy were identified using Youden’s index (J = sensitivity + specificity − 1). These thresholds are reported as exploratory and require external validation before clinical application.

Effect sizes for between-group differences were quantified using Cohen’s *d*. Multivariable logistic regression was performed to evaluate the independence of AGEs from conventional glycaemic markers, adjusting for age, sex, and HbA1c. Multiple linear regression was used to identify independent predictors of serum AGE concentration. Statistical significance was defined as a two-tailed *p* value below .05. All analyses were performed using Python 3.x with scipy, scikit-learn, and pandas libraries [14].

## Result

### Study population and baseline characteristics

A total of 226 participants were enrolled and allocated into five groups: healthy controls (HC, *n* = 37), isolated hyperlipidaemia (HL, *n* = 23), impaired glucose tolerance (IGT, *n* = 60), uncomplicated type 2 diabetes mellitus (T2DM, *n* = 54), and type 2 diabetes with complications (T2DM + C, *n* = 52). Demographic and clinical characteristics are summarized in Table S1, and biochemical parameters including lipid and renal profiles are presented in Table S2.

**Figure 1.**
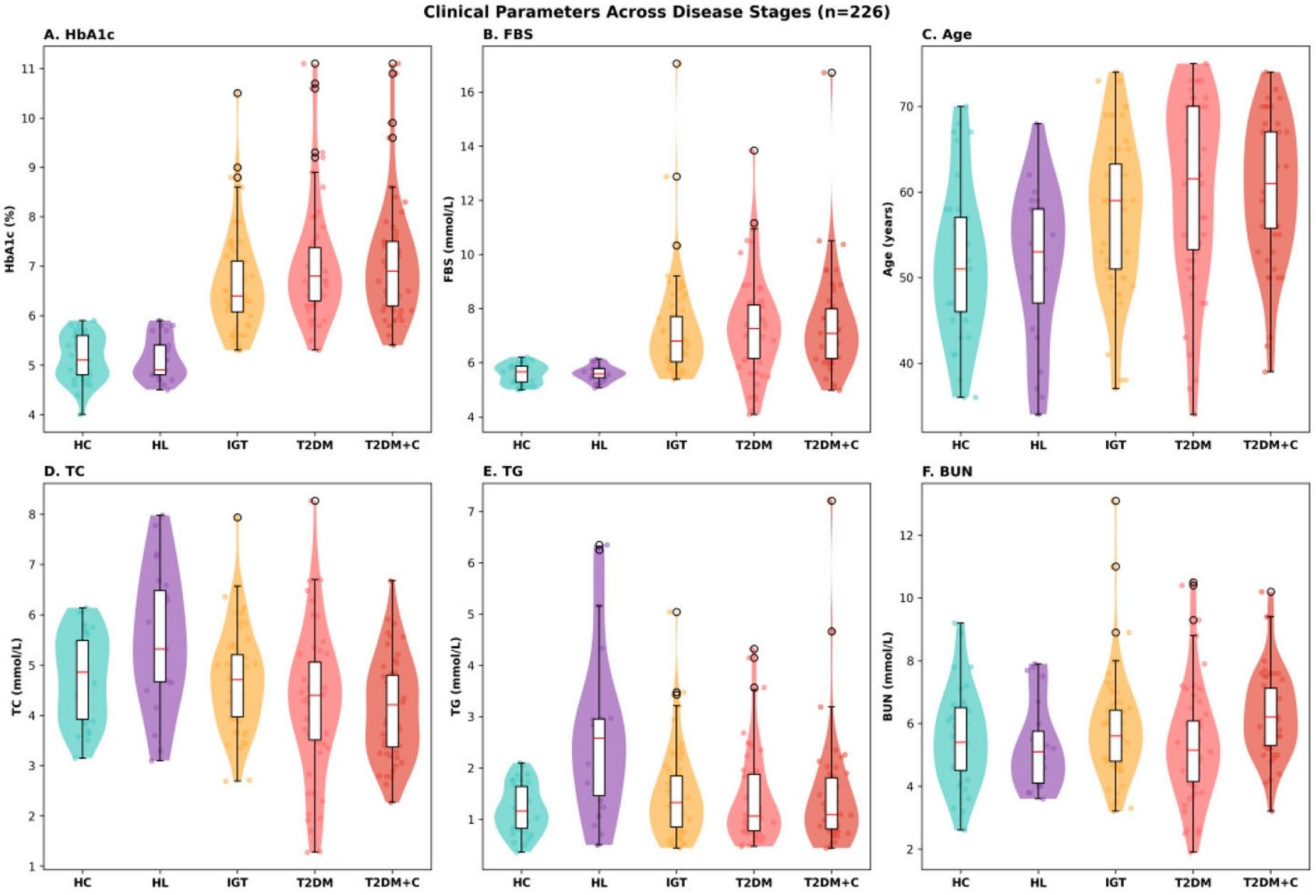
Distribution of clinical parameters across progressive disease stages. Clinical parameters were compared across five disease stages in 226 participants: healthy controls (HC), hyperlipidaemia (HL), impaired glucose tolerance (IGT), type 2 diabetes mellitus (T2DM), and T2DM with complications (T2DM + C). Violin plots with embedded box plots illustrate the distribution of each parameter, with individual data points overlaid as jittered dots. (A) Glycated haemoglobin (HbA1c) showed progressive elevation from HC (∼5%) through HL and IGT (∼6.5%), reaching highest levels in T2DM and T2DM + C (∼7%), with increased variance in advanced stages. (B) Fasting blood sugar (FBS) demonstrated marked stepwise increases, remaining normoglycemic in HC and HL (∼5.5 mmol/L), rising in IGT (∼7 mmol/L), and reaching diabetic ranges in T2DM and T2DM + C (∼7.5 mmol/L) with substantial inter-individual variability. (C) Age distributions indicated older populations in advanced disease stages, with median ages increasing from HC (∼50 years) to T2DM + C (∼60 years). (D) Total cholesterol (TC) was notably elevated in the HL group (∼5.5 mmol/L) compared to HC (∼4.8 mmol/L), with subsequent decreases in T2DM and T2DM + C (∼4.5 mmol/L). (E) Triglycerides (TG) showed the most pronounced elevation in HL (∼2.5 mmol/L), with high variability persisting through IGT and diabetic stages. (F) Blood urea nitrogen (BUN) remained relatively stable across groups, with slight increases in T2DM and T2DM + C and greater dispersion in advanced stages, potentially reflecting renal function variations. Colour coding is consistent across panels: HC (teal), HL (purple), IGT (yellow), T2DM (pink), and T2DM + C (red).

Mean age was comparable between HC (51.6 ± 8.9 years) and HL (51.7 ± 9.1 years) but was higher in the IGT (57.5 ± 8.8 years), T2DM (60.3 ± 10.6 years), and T2DM + C (60.1 ± 8.1 years) groups. Male representation increased progressively across the metabolic spectrum, from 35.1% (13/37) in HC to 56.5% (13/23) in HL, 51.7% (31/60) in IGT, 53.7% (29/54) in T2DM, and 71.2% (37/52) in T2DM + C. Glycaemic parameters demonstrated a stepwise escalation across groups: mean HbA1c rose from 5.13 ± 0.46% in HC and 5.12 ± 0.43% in HL to 6.64 ± 0.99% in IGT, 7.09 ± 1.25% in T2DM, and 7.11 ± 1.22% in T2DM + C, with a parallel pattern observed for fasting blood sugar, which increased from 5.59 ± 0.36 mmol/L in HC to 7.22 ± 1.88 mmol/L in IGT, 7.34 ± 1.84 mmol/L in T2DM, and 7.39 ± 1.85 mmol/L in T2DM + C (Table S1; Figure 1).

Among lipid parameters (Table S2), total cholesterol was highest in the HL group (5.52 ± 1.36 mmol/L) and tended to decline across the diabetic groups (IGT 4.71 ± 1.00, T2DM 4.24 ± 1.44, T2DM + C 4.15 ± 0.99 mmol/L). Triglycerides were markedly elevated in the HL group (2.64 ± 1.60 mmol/L) relative to HC (1.19 ± 0.47 mmol/L) and all other groups. HDL-cholesterol was lowest in the T2DM group (1.11 ± 0.43 mmol/L) compared with HC (1.38 ± 0.34 mmol/L), while LDL-cholesterol showed a declining trend from HC (2.54 ± 0.72 mmol/L) through T2DM + C (2.11 ± 0.72 mmol/L). Blood urea nitrogen was highest in the T2DM + C group (6.30 ± 1.37 mmol/L), consistent with the inclusion of patients with diabetic nephropathy, while uric acid did not differ meaningfully across groups (Table S2). The distributions of all measured biochemical parameters across study groups are presented in Supplementary Figure S3, and glycaemic progression patterns are detailed in Supplementary Figure S4.

### Serum AGE concentrations across the metabolic continuum

**Figure 2.**
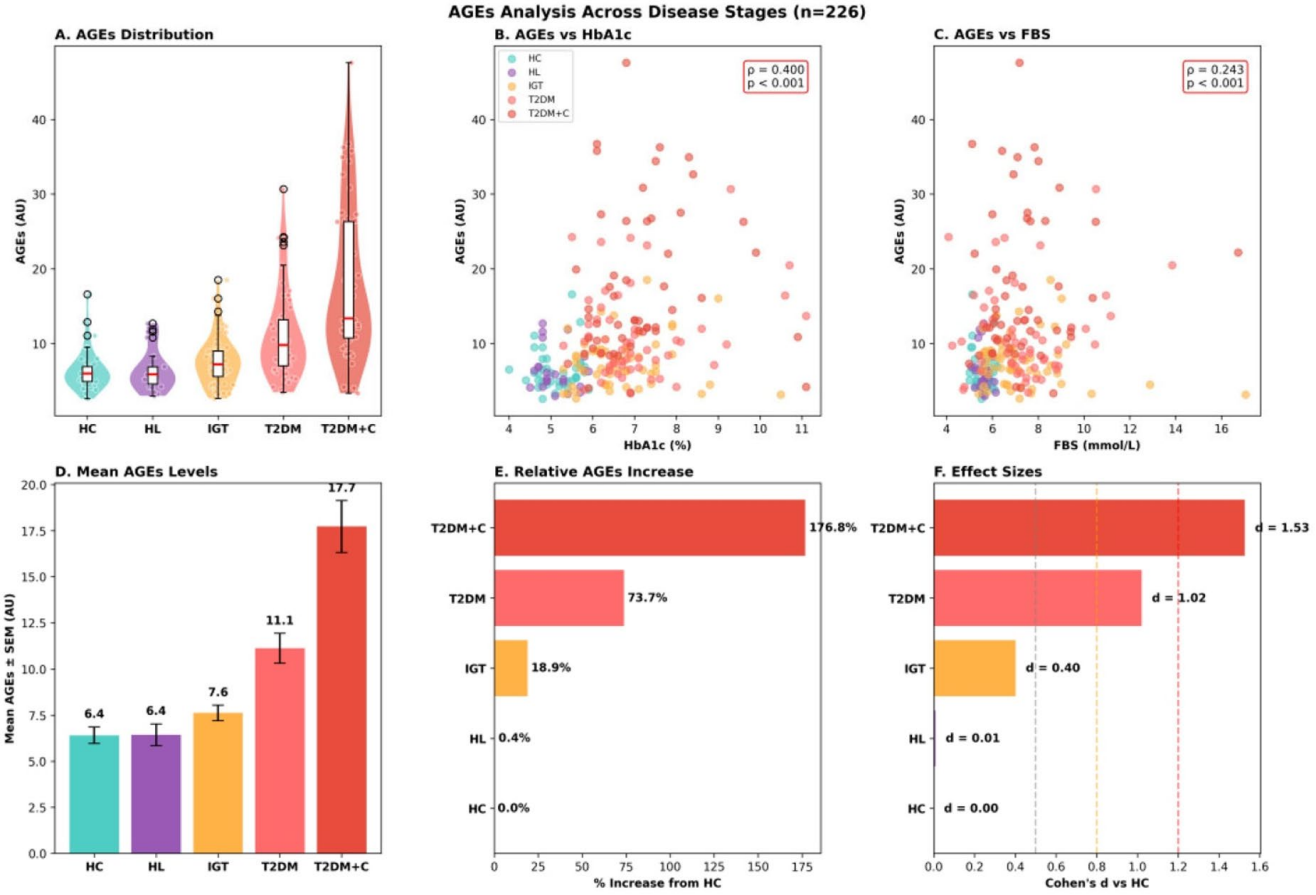
AGEs distribution across groups. (A) Violin plots with embedded box plots illustrate the distribution of AGEs levels (arbitrary units, AU) across disease stages, demonstrating progressively right-skewed distributions with increasing disease severity. (B,C) Scatter plots depict correlations between AGEs and glycaemic markers, revealing significant positive associations with HbA1c (ρ = 0.400, *p* < .001) and fasting blood sugar (FBS) (ρ = 0.243, *p* < .001). (D) Bar graph presenting mean AGEs ± SEM, showing stepwise elevation from HC (6.4 ± 0.4 AU) through HL (6.4 AU), IGT (7.6 AU), T2DM (11.1 AU), to T2DM + C (17.7 AU). (E) Waterfall plot quantifying relative AGEs increase compared to HC, with T2DM + C exhibiting a 176.8% increment. (F) Forest-style bar chart of Cohen’s *d* effect sizes versus HC, indicating negligible effects for HL (*d* = 0.01) and IGT (*d* = 0.40), large effects for T2DM (*d* = 1.02), and very large effects for T2DM + C (d = 1.53); dashed reference lines denote small (0.5), medium (0.8), and large (1.2) effect thresholds. Collectively, these data demonstrate that circulating AGEs accumulate progressively across the diabetes disease continuum, with the most pronounced elevation observed in complicated T2DM.

Median AGE levels were 5.99 (4.92–6.92) in HC, 5.86 (4.56–6.88) in HL, 7.22 (5.58–9.01) in IGT, 9.81 (7.04–13.20) in T2DM, and 13.35 (10.73–26.32) in T2DM + C (mean ± SD: 6.40 ± 2.71, 6.42 ± 2.82, 7.61 ± 3.27, 11.12 ± 5.95, and 17.72 ± 10.13 AU, respectively; Figure 2; Table S1).

Relative to healthy controls, AGE concentrations were essentially unchanged in the HL group (0.4% increase), showed a trend towards elevation in IGT (18.9% increase), were substantially elevated in uncomplicated T2DM (73.7% increase), and were markedly elevated in T2DM + C (176.8% increase). The near-identical AGE concentrations in the HL and HC groups (median 6.08 vs 6.12 AU; mean 6.42 ± 2.82 vs 6.40 ± 2.71 AU) demonstrate that isolated dyslipidaemia without concomitant glucose dysregulation does not contribute measurably to circulating AGE burden.

### Pairwise comparisons and effect sizes

**Figure 3.**
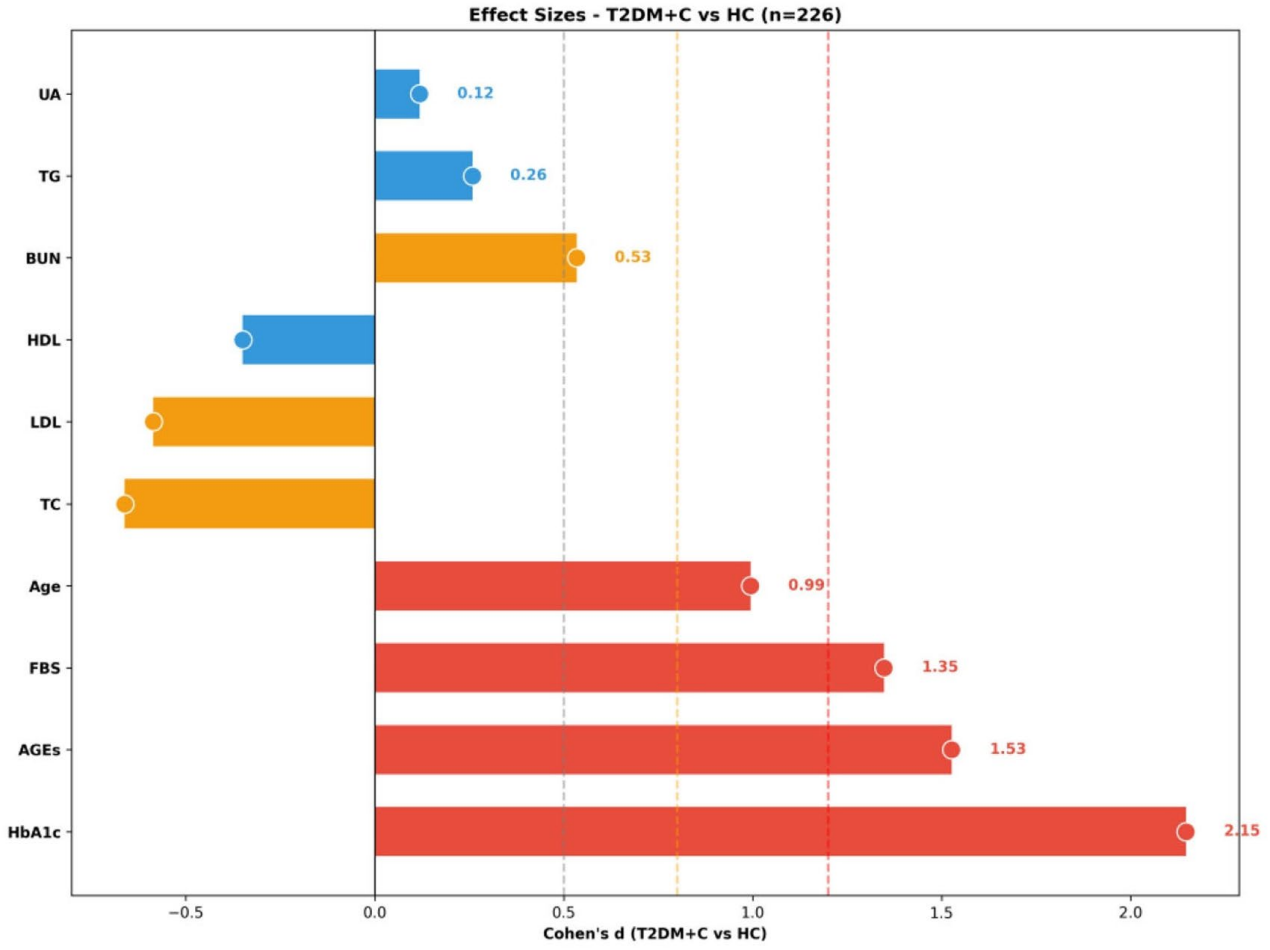
Comparative effect sizes of clinical parameters between T2DM with complications and healthy controls. Standardized effect sizes (Cohen’s *d*) were calculated to quantify the magnitude of differences between type 2 diabetes mellitus with complications (T2DM + C) and healthy controls (HC) across clinical parameters in 226 participants. The forest plot displays horizontal bars representing Cohen’s *d* values, with positive effects (red/orange bars) indicating higher values in T2DM + C and negative effects (blue bars) indicating lower values in T2DM + C relative to HC. Vertical reference lines denote conventional effect size thresholds: small (*d* = 0.5, dashed gray), medium (*d* = 0.8, dashed orange), and large (*d* = 1.2, dashed red). Glycaemic markers demonstrated the largest effect sizes, with HbA1c showing the strongest difference (*d* = 2.15, very large effect), followed by AGEs (*d* = 1.53, very large effect) and fasting blood sugar (*d* = 1.35, large effect). Age exhibited a large effect size (*d* = 0.99), consistent with older populations in complicated diabetes. Lipid parameters showed divergent patterns: total cholesterol (TC) and low-density lipoprotein (LDL) were decreased in T2DM + C (*d* ≈ −0.6), while high-density lipoprotein (HDL) showed a moderate negative effect. Triglycerides (TG) and uric acid (UA) displayed small positive effects (*d* = 0.26 and 0.12, respectively), whereas blood urea nitrogen (BUN) showed a moderate positive effect (*d* = 0.53). These findings highlight that glycaemic indicators and AGEs demonstrate the most pronounced differences between complicated diabetes and healthy states, exceeding traditional cardiometabolic risk factors in discriminatory magnitude

Post-hoc pairwise comparisons with effect size estimation are presented in full in Table S3 and visualized in Figure 3. For serum AGEs, all comparisons between groups with differing glycaemic status achieved statistical significance after Bonferroni correction except HC versus IGT.

The HC versus IGT comparison yielded a small-to-medium effect size (uncorrected *p* = .037, Cohen’s *d* = 0.425) but did not remain significant after Dunn’s test with Bonferroni correction (*p* = .094).

The HC versus T2DM (*p* < .001, *d* = 1.027) and HC versus T2DM + C (*p* < .001, *d* = 1.540) both demonstrated large effect sizes. The comparison between IGT and T2DM was significant with a medium effect (*p* < .001, *d* = 0.721), and IGT versus T2DM + C showed a large effect (*p* < .001, *d* = 1.346). The transition from uncomplicated to complicated diabetes remained highly significant (*p* < .001, *d* = 0.802), indicating that complication development is accompanied by a substantial further acceleration in AGE accumulation (59.4% increase beyond uncomplicated T2DM).

A particularly noteworthy pattern emerged when AGE pairwise comparisons were contrasted with those of conventional glycaemic markers (Table S3). While HbA1c sharply differentiated HC from all disease groups (*d* = 1.961–2.182, all *p* < .001), it failed to discriminate between the diabetic subgroups: IGT versus T2DM yielded only a small effect (*d* = 0.357, *p* = .046), and T2DM versus T2DM + C showed virtually no difference (*d* = 0.022, *p* = .706). Fasting blood sugar displayed an even more pronounced plateau, with no significant differences among IGT, T2DM, and T2DM + C (all *p* > .33, *d* ≤ 0.030). In contrast, serum AGEs continued to differentiate significantly at every stage transition except HC to IGT after strict Bonferroni correction, including between T2DM and T2DM + C (*d* = 0.802, *p* < .001), demonstrating discriminative capacity precisely where conventional glycaemic markers plateau (Table S3; Figure 3).

### AGE concentrations and complication burden

**Figure 4.**
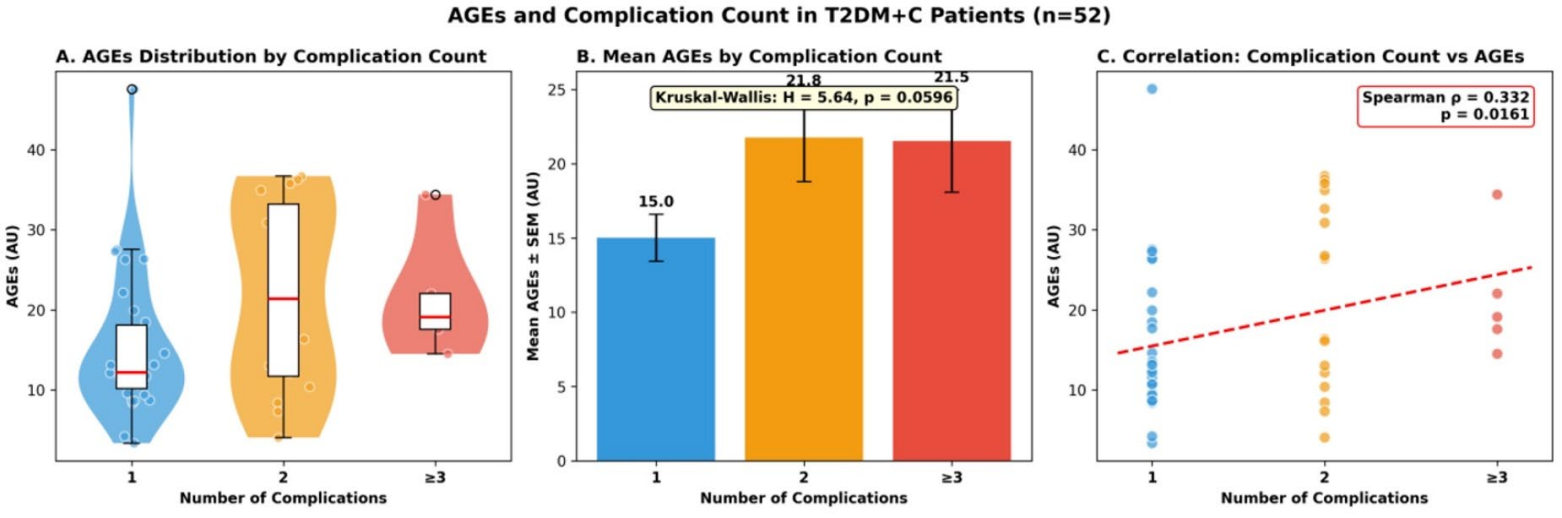
Association between AGEs levels and complication burden in T2DM + C patients. The relationship between Advanced Glycation End-products (AGEs) and the number of diabetes complications was examined in 52 patients with type 2 diabetes mellitus and complications (T2DM + C). Patients were stratified by complication count: 1 complication (blue), 2 complications (orange), and ≥3 complications (red). (A) Violin plots with embedded box plots illustrate AGEs distributions across complication burden groups. The single complication group displayed the widest distribution with several high outliers (up to ∼45 AU), while the ≥3 complications group showed a more constrained, elevated distribution. (B) Bar graph presenting mean AGEs ± SEM, revealing a stepwise increase from 15.0 ± 1.6 AU in patients with 1 complication to 21.8 ± 2.9 AU in those with two complications, with a slight plateau at 21.5 ± 3.4 AU in patients with ≥3 complications. The Kruskal–Wallis test indicated a marginally significant difference across groups (*H* = 5.64, *p* = .0596). (C) Scatter plot with linear regression demonstrates a significant positive correlation between complication count and AGEs levels (Spearman ρ = 0.332, *p* = .0161), with the fitted trend line indicating progressive AGEs accumulation with increasing complication burden. These findings suggest that higher circulating AGEs are associated with greater polycomorbidity in diabetic patients, supporting the potential utility of AGEs as a biomarker for monitoring cumulative microvascular and macrovascular damage.

Within the T2DM + C group, complication burden was distributed as follows: 31 patients (59.6%) had a single complication, 16 patients (30.8%) had two complications, and 5 patients (9.6%) had three or more complications (Table S4). AGE concentrations demonstrated a dose–response relationship with increasing complication count: patients with a single complication had mean AGE levels of 15.02 ± 8.80 AU, those with two complications had 21.77 ± 11.86 AU, and those with three or more complications had 21.53 ± 7.69 AU (Table S4; Figure 4). Spearman correlation analysis confirmed a significant positive association between complication count and serum AGE concentration (ρ = 0.332, *p* = .016). Importantly, this dose–response pattern was specific to AGEs: neither HbA1c (7.18 ± 1.41%, 6.95 ± 0.95%, and 7.22 ± 0.72% for one, two, and three or more complications, respectively) nor fasting blood sugar (7.54 ± 2.11, 7.21 ± 1.51, and 7.00 ± 1.07 mmol/L) showed any meaningful escalation with complication count (Table S4), further supporting the concept that AGEs capture a dimension of cumulative metabolic injury not reflected by conventional glycaemic markers.

The plateau observed between the two-complication and three-or-more-complication subgroups (21.77 versus 21.53 AU) should be interpreted cautiously given the small sample size in the latter category (*n* = 5).

### Correlation analysis

Spearman rank correlation analysis was performed to evaluate associations between serum AGEs and clinical parameters across the entire study population (n = 226). Individual bivariate scatter plots are shown in Supplementary Figure S2, with category-specific correlation matrices in Supplementary Figures S5–S8 and a comprehensive pairwise overview in Supplementary Figure S9.

HbA1c emerged as the strongest single correlate of serum AGEs (ρ = 0.428, *p* < .001), followed by fasting blood sugar (ρ = 0.268, *p* < .001), confirming the glucose-dependent nature of AGE formation (Supplementary Figure S5). The squared correlation coefficient (r² ≈ 0.18) indicates that HbA1c alone explains approximately 18% of the variance in serum AGEs, highlighting substantial independence between these biomarkers and suggesting that AGEs capture cumulative metabolic damage not fully reflected by medium-term glycemic averaging. Age showed a weak positive correlation (ρ = 0.140, p = 0.019).

Among lipid parameters (Supplementary Figure S6) total cholesterol demonstrated a modest but statistically significant inverse correlation with AGEs (ρ = −0.176, 0.003),whereas triglycerides showed no meaningful association (ρ = −0.001, p = 0.983). No substantial correlations were observed between AGEs and remaining lipid parameters. Renal parameters, including blood urea nitrogen (ρ = 0.091, p = 0.127) and uric acid (ρ = 0.01), showed no significant associations with serum AGEs (Supplementary Figure S7). Hepatic enzymes were similarly uncorrelated with AGEs (ALT: ρ = 0.03; AST: ρ = −0.02) (Supplementary Figure S8).

To assess robustness to age-related confounding, partial Spearman correlations controlling for age were computed. The AGEs-HbA1c correlation remained significant (ρ = 0.396, *p* < .001), as did the AGEs-FBS correlation (ρ = 0.219, *p* < .001), confirming that these associations are not merely driven by the older age of diabetic participants (Supplementary Figure S5).

### Multivariable analyses

To establish whether AGEs provide independent information beyond conventional glycaemic markers, we performed multivariable logistic regression for discriminating T2DM + C from HC. After adjustment for age, sex, and HbA1c, serum AGEs remained a significant independent predictor (adjusted OR = 4.92, 95% CI [2.98–8.98]). HbA1c also remained strongly predictive (adjusted OR = 14.65, 95% CI [9.64–21.21]), as did age (adjusted OR = 1.94, 95% CI [1.21–3.16]). and male sex (OR = 1.90, 95% CI [1.21–2.99]).The model demonstrated excellent discrimination (10-fold cross-validated AUC = 0.971 ± 0.024), (Table S7a). These findings establish that AGEs contribute non-redundant discriminative information and are best utilized as complementary biomarkers alongside HbA1c and clinical risk factors.

Multiple linear regression with serum AGEs as the dependent variable identified HbA1c as the strongest independent predictor (β = 2.756), followed by male sex (β = 2.015) and LDL-cholesterol (β = −1.554), with an overall model R^2^ of 0.168. Notably, fasting blood sugar was negatively associated with AGEs (β = −0.804) in the multivariable model, likely reflecting multicollinearity with HbA1c (ρ = 0.80). (Table S7b).

### Diagnostic performance of serum AGEs

**Figure 5.**
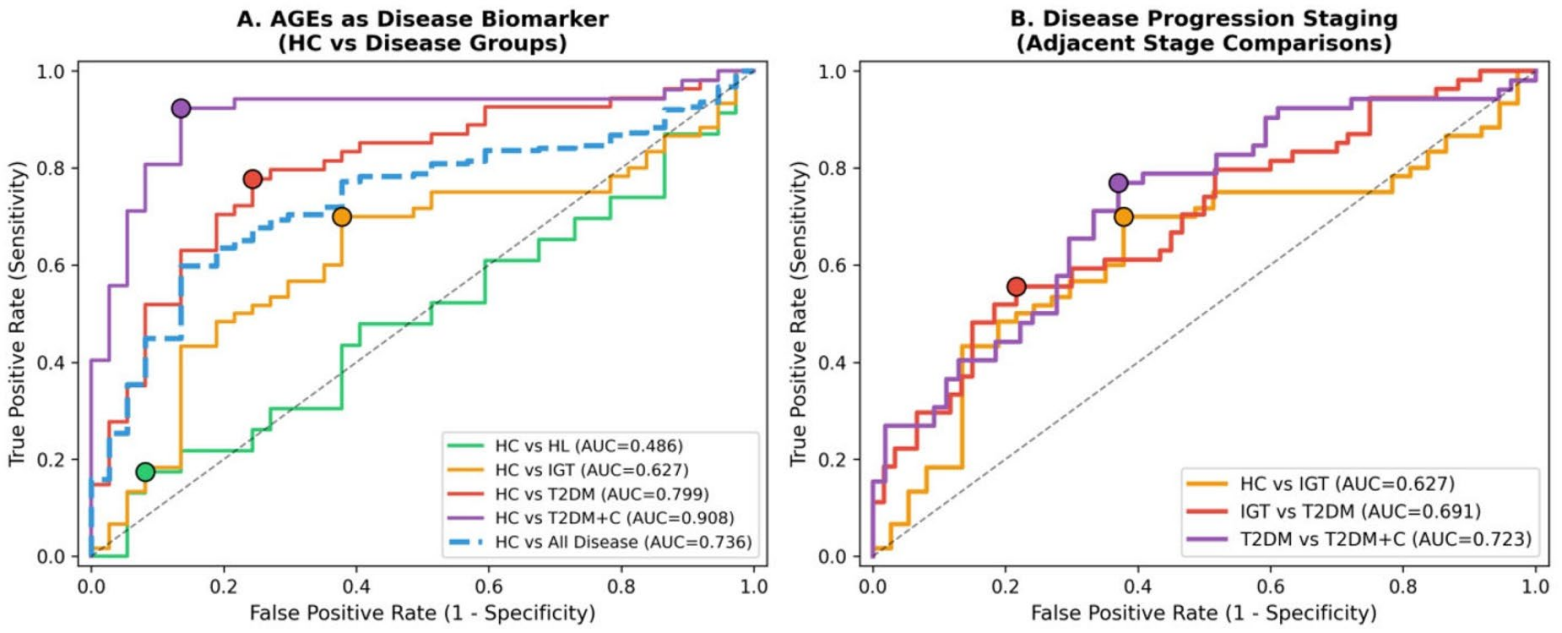
Receiver operating characteristic (ROC) analysis of AGEs for disease discrimination. The diagnostic performance of Advanced Glycation End-products (AGEs) was evaluated using ROC curve analysis across different disease comparisons. (A) ROC curves comparing healthy controls (HC) against individual disease groups and all disease groups combined. AGEs demonstrated excellent discriminatory capacity for distinguishing HC from T2DM with complications (T2DM + C) (AUC = 0.908, purple curve), good performance for HC vs T2DM (AUC = 0.799, red curve), and fair performance for HC vs all disease groups combined (AUC = 0.736, dashed blue curve). In contrast, AGEs showed poor discrimination between HC and hyperlipidaemia (HL) (AUC = 0.486, green curve) and modest discrimination for HC vs impaired glucose tolerance (IGT) (AUC = 0.627, orange curve). Optimal cutoff points (marked by coloured circles) were determined using Youden’s index. (B) ROC curves for adjacent disease stage comparisons to assess AGEs utility in monitoring disease progression. AGEs showed modest discriminatory ability between HC and IGT (AUC = 0.627, orange curve), between IGT and T2DM (AUC = 0.691, red curve), and between T2DM and T2DM + C (AUC = 0.723, purple curve), suggesting incremental value in tracking disease advancement. The diagonal dashed line represents the reference line of no discrimination (AUC = 0.500). Collectively, these data indicate that AGEs serve as a robust biomarker for detecting established diabetes and its complications, with limited utility for distinguishing early metabolic abnormalities from healthy states.

Receiver operating characteristic analysis was performed to evaluate the discriminative capacity of serum AGEs in two complementary frameworks: biomarker validation against healthy controls (Table S6) and clinical staging across sequential disease transitions (Table S5). All ROC curves are presented in Figure 5.

In the biomarker validation analysis (Table S6), serum AGEs achieved the highest diagnostic accuracy for distinguishing T2DM + C from HC (AUC = 0.908, 95% CI [0.834–0.970]; sensitivity 92.3%; specificity 86.5%; optimal threshold 8.394 AU), representing excellent discriminative performance. Clinically meaningful discrimination was also observed for T2DM versus HC (AUC = 0.799, 95% CI [0.705–0.888]; sensitivity 77.8%; specificity 75.7%; threshold 6.993 AU) and for IGT versus HC (AUC = 0.627, 95% CI [0.512–0.736]; sensitivity 70.0%; specificity 62.2%; threshold 6.265 AU). As expected, AGEs showed no discriminative value for distinguishing HL from HC (AUC = 0.486, 95% CI [0.331–0.643]), consistent with the absence of AGE elevation in isolated dyslipidaemia. When all disease groups were pooled, AGEs discriminated any metabolic disease from healthy controls with moderate accuracy (AUC = 0.736, 95% CI [0.642–0.827]; sensitivity 59.8%; specificity 86.5%; threshold 8.014 AU).

In the sequential clinical staging analysis (Table S5), which evaluates AGE performance at each successive disease transition, the HC to IGT transition yielded an AUC of 0.627 (95% CI [0.512–0.736]), the IGT to T2DM transition yielded an AUC of 0.691 (95% CI [0.587–0.782]), and the T2DM to T2DM + C transition yielded an AUC of 0.723 (95% CI [0.626–0.817]). The progressive increase in optimal thresholds across successive disease stages – from 6.3 to 9.1 to 10.7 AU – mirrors the stepwise AGE accumulation observed in the group-level analysis. These thresholds are provided as proof-of-concept only; their clinical applicability is currently limited by the modest AUCs for early-stage transitions and the need for prospective external validation.

## Discussion

The present study provides one of the most comprehensive cross-sectional characterizations of circulating AGE concentrations across the full metabolic disease trajectory, spanning normoglycaemia, isolated dyslipidaemia, prediabetes, uncomplicated type 2 diabetes, and diabetes complicated by end-organ damage. Four principal findings emerge from this investigation that advance our understanding of AGE biology in metabolic disease. First, serum AGEs accumulate in a progressive, stepwise fashion across the metabolic continuum, with each successive disease stage demonstrating quantitatively greater AGE burden. Second, this accumulation is detectable at the prediabetic stage, before conventional diagnostic thresholds for diabetes are met, suggesting that non-enzymatic glycation commences early in the natural history of glucose dysregulation. Third, isolated hyperlipidaemia, despite generating substantial oxidative stress, does not produce measurable AGE elevation, thereby reinforcing the mechanistic primacy of hyperglycaemia in driving non-enzymatic glycation reactions. Fourth, serum AGEs maintain significant discriminative capacity across the full disease trajectory, including the clinically critical transition from uncomplicated to complicated diabetes (AUC = 0.723, Cohen’s *d* = 0.802), precisely where conventional glycaemic markers such as HbA1c (*d* = 0.022) and fasting blood sugar (*d* = 0.023) plateau, positioning AGE measurement as a candidate complementary biomarker for complication risk stratification in established diabetes.

## Progressive AGE accumulation: magnitude and mechanisms

The graded elevation from 6.40 ± 2.71 AU in controls to 17.72 ± 10.13 AUin complicated T2DM recapitulates the established understanding that AGE formation follows second-order kinetics dependent on both sugar concentration and exposure duration [6,15]. The effect sizes were substantial: uncomplicated diabetes showed a 73.7% increase over controls (Cohen’s *d* = 1.027), while complicated diabetes exhibited a 176.9% increase (*d* = 1.540), exceeding those typically reported for conventional biochemical parameters across the same disease stages.

The disproportionate further elevation in complicated disease (an additional 59.4% beyond uncomplicated T2DM) likely reflects the convergence of multiple amplification loops rather than hyperglycaemia alone. Progressive nephropathy impairs the renal clearance of free AGE adducts, establishing a vicious cycle in which renal damage promotes AGE retention and further renal injury [16]. Concurrently, heightened oxidative stress amplifies the generation of reactive dicarbonyl intermediates methylglyoxal, glyoxal, and 3-deoxyglucosone which are orders of magnitude more reactive than glucose in forming AGE adducts [17,18]. The saturation of glyoxalase detoxification under sustained metabolic stress further compounds this self-amplifying cascade [18]. These findings are concordant with the Veterans Affairs Diabetes Trial, in which Koska and colleagues demonstrated that baseline plasma AGEs independently predicted incident cardiovascular events after comprehensive adjustment for conventional risk factors including HbA1c [19]. and with the longitudinal data of Saulnier et al. [20]. showing that AGE concentrations predicted subsequent renal function loss and correlated with histopathological severity of diabetic kidney disease.

## Early accumulation in prediabetes

Among the most clinically significant observations is the trend towards AGE elevation in individuals with impaired glucose tolerance (18.9% increase; uncorrected *p* = .037, Bonferroni-corrected *p* = .094), indicating that non-enzymatic glycation may already be operative during the prediabetic phase. This period, characterized by intermittent postprandial hyperglycaemic excursions and incipient insulin resistance, provides sufficient substrate for Maillard chemistry even when fasting glucose remains within normal limits [15,21]. The finding extends prior evidence from the LifeLines cohort, in which van Waateringe and colleagues demonstrated that skin autofluorescence, a non-invasive tissue AGE proxy, independently predicted incident T2DM over four years of follow-up [22]. Emerging evidence further suggests that dicarbonyl stress from impaired glyoxalase activity may represent an early metabolic disturbance in insulin-resistant states that precedes β-cell failure [18,23]. Uribarri et al. similarly reported elevated serum AGEs in obese individuals meeting metabolic syndrome criteria compared with metabolically healthy obese controls [24]. Collectively, these data support AGE formation as an early biochemical footprint of metabolic deterioration, detectable during the critical prediabetic window when intervention has the greatest potential to alter disease trajectory.

## Specificity for glycaemic dysregulation

The inclusion of an isolated hyperlipidaemia group without glucose dysregulation enabled direct interrogation of whether dyslipidaemia contributes independently to AGE accumulation. AGE concentrations in this group were virtually identical to healthy controls (median 6.08 vs 6.12 AU; mean 6.42 ± 2.82 vs 6.40 ± 2.71 AU, *p* = .987). While lipid peroxidation generates reactive carbonyl species that modify proteins through analogous chemistry, these reactions proceed through pathways chemically and structurally distinct from the glucose-dependent Maillard reaction [25]. Moldogazieva and colleagues have emphasized that despite shared downstream signalling consequences including RAGE binding and NF-κB activation the precursor chemistry of AGEs and advanced lipoxidation end products differs fundamentally.

An important caveat must be acknowledged i.e. our ELISA-based measurement approach captures immunoreactive AGE species recognized by the assay antibody and may not detect structurally distinct adducts such as Nε-(carboxymethyl)lysine, which can be generated through both glycoxidation and lipoxidation pathways [6,18]. Future mass spectrometry-based studies would be needed to fully delineate pathway-specific contributions to the heterogeneous AGE pool.

## Correlation architecture and complementarity to HbA1c

HbA1c emerged as the strongest single correlate of serum AGEs (ρ = 0.400), yet explained only approximately 16% of AGE variance. This substantial independence is mechanistically expected: HbA1c reflects mean glycaemia over approximately 120 days on a single protein in a single compartment, whereas AGEs accumulate on diverse long-lived substrates over timescales of weeks to decades and are additionally influenced by dicarbonyl generation, glyoxalase efficiency, and oxidative stress none of which is captured by HbA1c [15,17,18]. Total cholesterol demonstrated a modest but statistically significant inverse correlation with AGEs (ρ = −0.146, *p* = .029), while HDL-cholesterol showed no significant association (ρ = 0.003, *p* = .964). The inverse relationship with total cholesterol may reflect confounding by statin use in diabetic groups or alterations in lipoprotein metabolism associated with advanced glycation [26]. These data suggest that AGEs capture dimensions of cumulative metabolic injury not reflected by conventional glycaemic averaging.

The capacity of AGEs to differentiate between disease stages was most strikingly demonstrated by contrasting AGE and HbA1c performance across the metabolic spectrum. While HbA1c powerfully discriminated healthy controls from all disease groups (Cohen’s *d* = 1.961–2.182), it showed virtually no discriminative capacity between uncomplicated and complicated diabetes (*d* = 0.022, *p* = .706). Fasting blood sugar displayed a similarly complete plateau across diabetic groups (*d* = 0.023, *p* = .908). In contrast, serum AGEs continued to differentiate significantly at the T2DM-to-T2DM + C transition (*d* = 0.802, *p* < .001), providing clinically relevant discrimination precisely where conventional markers are uninformative.

Our demonstration that AGE elevation is detectable in prediabetes and accumulates progressively supports the hypothesis that AGE measurement may serve as a direct quantitative index of metabolic memory, capturing cumulative glycaemic burden that HbA1c cannot.

It is critical to distinguish association from prediction. The cross-sectional design precludes any inference regarding the predictive value of AGEs for future complication risk. The observed correlations and ROC-derived thresholds reflect cross-sectional associations that require prospective validation. We therefore frame AGEs as a complementary biomarker reflecting cumulative glycaemic burden a dimension biochemically distinct from HbA1c rather than a replacement for established glycaemic monitoring.

## Strengths and limitations

This study benefits from the inclusion of five clinically defined groups spanning the complete metabolic trajectory, standardized pre-enrolment dietary screening to mitigate confounding from exogenous AGE intake [24,27]. The incorporation of an isolated hyperlipidaemia group as a metabolic control enabled direct assessment of the specificity of AGE elevation for glycaemic versus lipid-mediated metabolic stress, a question that has not been systematically addressed in the existing literature. The use of standardized pre-enrolment dietary screening to exclude participants with unusual dietary patterns mitigated a recognized source of confounding, given the established contribution of dietary AGE intake to circulating AGE concentrations. The comprehensive biochemical profiling of all participants, encompassing glycaemic, lipid, hepatic, and renal parameters, facilitated multivariable correlation analyses that delineated the relationship between AGEs and conventional clinical markers across the entire study population. Furthermore, the rigorous application of non-parametric statistical methods appropriates to the distributional characteristics of the data, combined with Bonferroni correction for multiple comparisons, strengthens confidence in the reported between-group differences.

Despite the clear trends observed, certain limitations warrant consideration. The cross-sectional design precludes definitive conclusions regarding the causal relationship between AGE accumulation and the progression of complications. Additionally, while **ELISA** is a widely used and practical method for AGE quantification, it is limited to AGE epitopes recognized by the assay antibody and may not capture the full spectrum of structurally diverse AGE species, such as carboxymethyl-lysine (CML). The ELISA-based approach captures only a fraction of the structurally heterogeneous AGE pool. Future studies employing LC-MS/MS or high-resolution metabolomics are needed to delineate which specific AGE species drive the observed associations and to validate the clinical utility of total immunoreactive AGE measurement.

The unequal sex distribution across groups, with male predominance increasing from 35.1% in healthy controls to 71.2% in complicated diabetes, introduces a potential confounding variable given reported sex differences in AGE metabolism and RAGE expression. Finally, the single-centre design and the specific demographic characteristics of the Shaoxing population may limit the generalizability of our findings to other ethnic, geographic, and healthcare settings, and multi-centre validation across diverse populations will be important to confirm the universality of the patterns observed.

An important alternative interpretation is that AGE elevation in T2DM + C reflects reduced renal clearance rather than increased production. While serum creatinine and BUN were measured, eGFR was not formally calculated, and the modest correlation between BUN and AGEs (ρ = 0.028, *p* = .678) does not support renal impairment as the primary driver. Nevertheless, we cannot exclude reduced clearance as a contributory factor, particularly in patients with diabetic nephropathy.

The absence of detailed medication records including anti-diabetic agents (metformin, sulfonylureas, SGLT2 inhibitors, GLP-1 receptor agonists, insulin), statins, ACE inhibitors, and antihypertensives represents a significant limitation, as these medications may influence both AGE levels and complication risk. Similarly, the lack of systematic recording of dietary supplement use, smoking status, and physical activity prevents comprehensive confounding adjustment. The absence of inflammation markers (CRP, IL-6, TNF-α) and oxidative stress markers (malondialdehyde, advanced oxidation protein products) further limits mechanistic interpretation.

The modest sample size (*n* = 226), unequal group sizes, and single-centre design limit statistical power and generalizability. These findings should be interpreted as hypothesis-generating rather than definitive, and require validation in larger, multi-ethnic cohorts. The stage-specific cut-offs (6.3, 9.1, and 10.7 AU) are provided as proof-of-concept only. Their clinical applicability is currently limited by the modest AUCs for early-stage transitions (e.g. HC vs IGT: AUC = 0.627, 95% CI [0.512–0.736]), the small sample size, and the single-centre design. Although non-parametric methods were used for primary analyses, supplementary parametric descriptors (mean ± SD) and Cohen’s d effect sizes were additionally reported for comparability with prior literature and should be interpreted cautiously given the skewed AGE distributions. Furthermore, multicollinearity between HbA1c and fasting blood sugar (ρ = 0.80) limits independent interpretation of their regression coefficients. ROC-derived thresholds were internally estimated within a cross-sectional cohort and should therefore be considered exploratory pending prospective external validation

## Future directions

The findings of this study establish a foundation for several investigative priorities. Prospective multicentre cohort studies incorporating serial AGE measurements at standardized intervals are needed to determine whether AGE trajectories can predict incident complications with sufficient accuracy to influence clinical decision-making. The application of liquid chromatography-tandem mass spectrometry for quantification of individual AGE molecular species would enable species-specific clinical associations and may reveal differential pathogenic roles for specific compounds. Integration of AGE measurements with other emerging biomarkers, including soluble RAGE, dicarbonyl metabolites, and markers of oxidative stress, within multi-analyte panels warrants evaluation for enhanced risk prediction accuracy. Finally, interventional studies examining whether AGE-lowering strategies, whether pharmacological, dietary, or lifestyle-based, translate into meaningful reduction in complication risk would be essential to establish AGEs not only as biomarkers but as modifiable therapeutic targets.

## Supplementary Material

Supplemental Material

## Data Availability

The data that support the findings of this study are available from the corresponding author(Xinyun Zhang) upon reasonable request. The data are not publicly available due to privacy or ethical restrictions.

## Open scholarship

This article has earned the Center for Open Science badges for Open Materials. The materials are openly accessible at 10.6084/m9.figshare.32098231.
