# Supplementary material for: Serum advanced glycation end-products as a biomarker of cumulative glycaemic burden and complications in type 2 diabetes: a pilot study
Source: Ann Med. 2026 May 20;58(1):2671517. doi: 10.1080/07853890.2026.2671517 (PMC13195711; doi:10.1080/07853890.2026.2671517)
Supplement: Supplemental Material [file IANN_A_2671517_SM2623.docx]

# Supplementary

## Figure S1. Z-Score Normalized Heatmap of Clinical Parameters Across Disease Stages


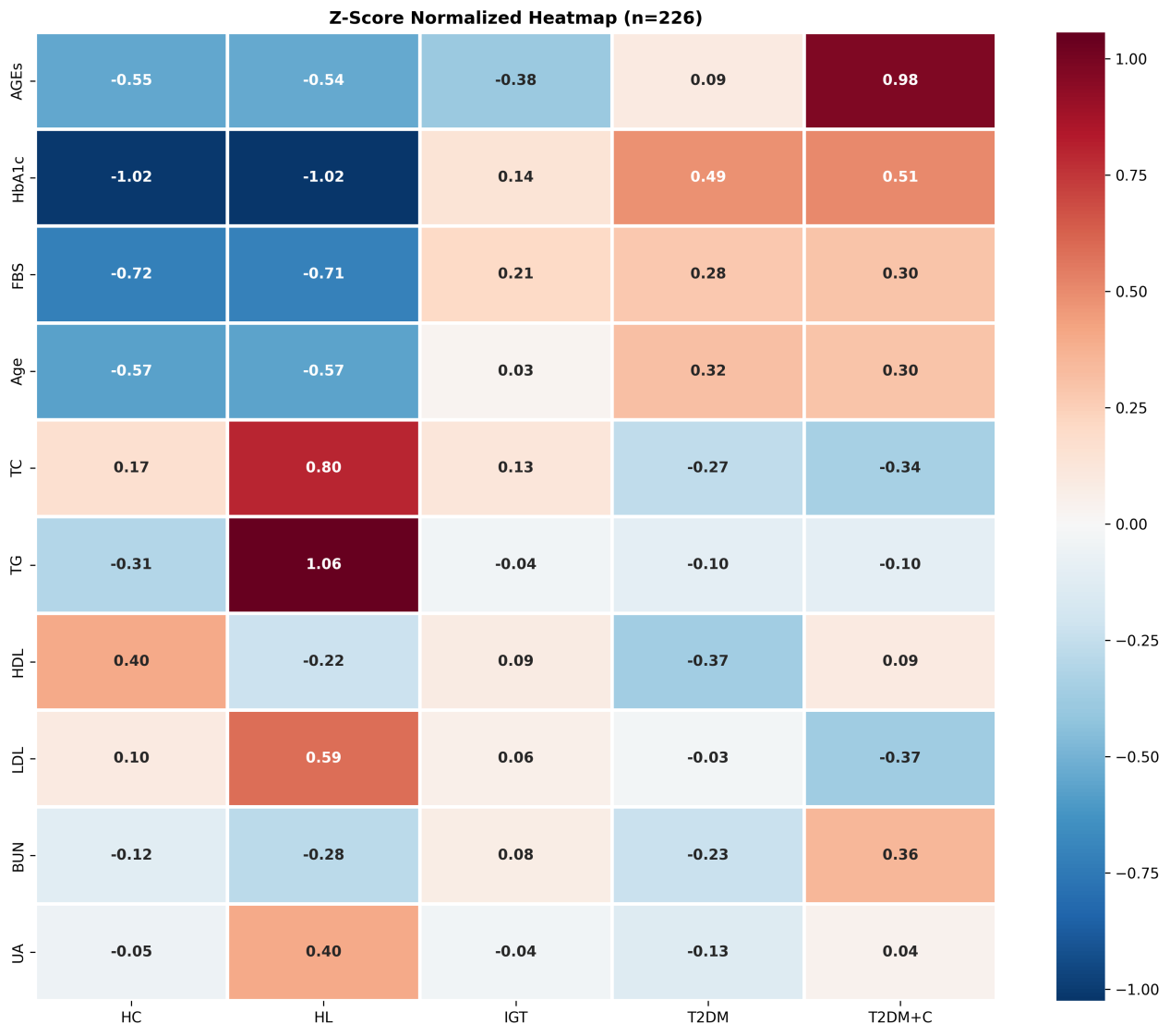


*Heatmap displaying standardized mean values (Z-scores) of 10 clinical parameters across five disease groups in 226 participants. Z-scores were calculated relative to the overall cohort mean, with red hues indicating values above the mean and blue hues indicating values below the mean. Columns represent disease stages: healthy controls (HC), hyperlipidemia (HL), impaired glucose tolerance (IGT), type 2 diabetes mellitus (T2DM), and T2DM with complications (T2DM+C). Notable patterns include: (1) progressive elevation of glycemic markers (HbA1c, FBS) and AGEs from HC through T2DM+C, with AGEs showing the steepest gradient (Z-score: -0.55 in HC to +0.98 in T2DM+C); (2) marked elevation of lipid parameters (TC, TG, LDL) specifically in the HL group, with TG showing the highest Z-score (1.06); (3) relatively stable age distribution across groups with modest increases in diabetic stages; and (4) divergent patterns for HDL, with elevation in HC and depletion in T2DM. This visualization highlights distinct metabolic signatures characterizing each disease stage, with AGEs demonstrating the most pronounced disease-associated elevation among all parameters examined.*

## Figure S2. Bivariate Correlations Between AGEs and Key Clinical Parameters


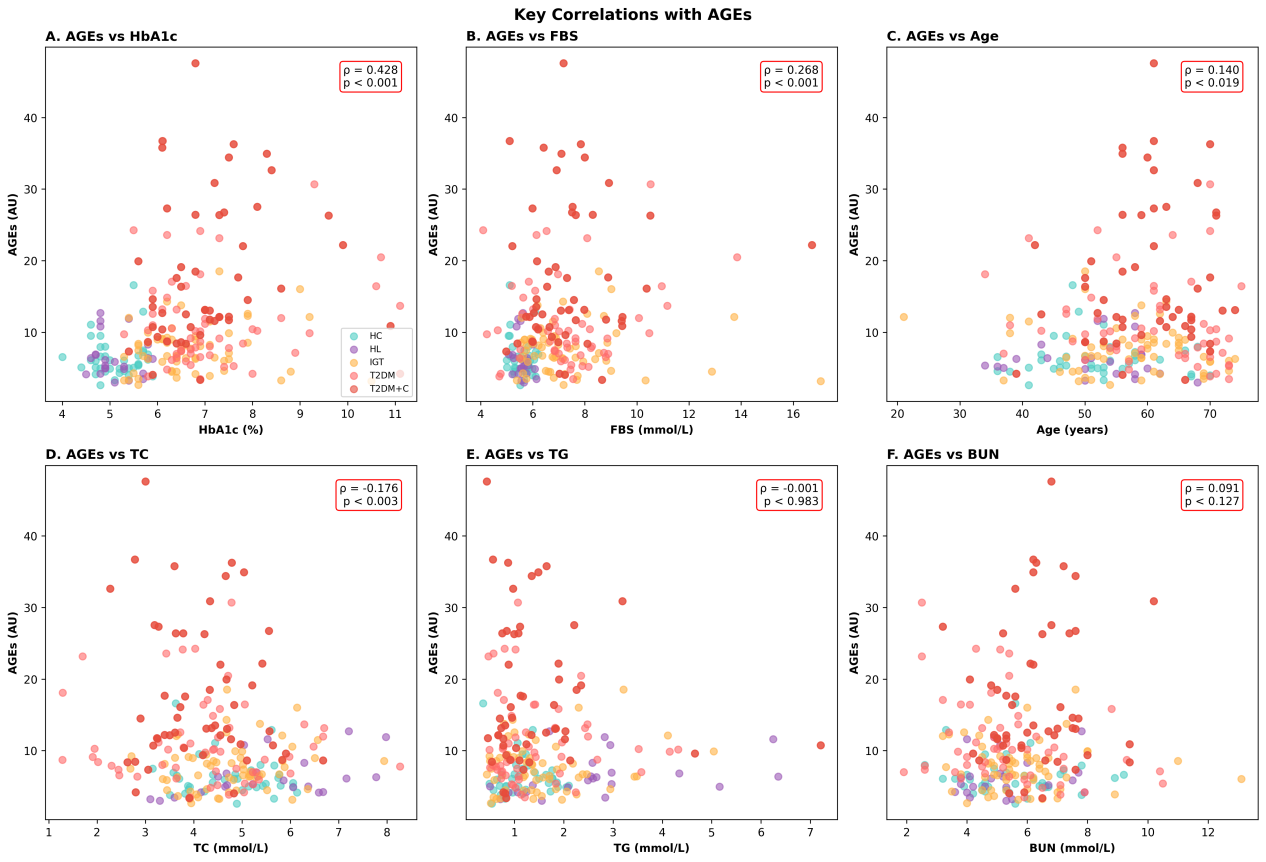


*Scatter plots illustrating the relationships between Advanced Glycation End-products (AGEs) and six clinical variables in 226 participants across five disease groups: healthy controls (HC, teal), hyperlipidemia (HL, purple), impaired glucose tolerance (IGT, yellow), type 2 diabetes mellitus (T2DM, pink), and T2DM with complications (T2DM+C, red). Spearman correlation coefficients (ρ) and p-values are inset for each panel.* ***(A)*** *AGEs vs HbA1c: moderate positive correlation (ρ = 0.428, p < 0.001) with clear separation of disease groups.* ***(B)*** *AGEs vs fasting blood sugar (FBS): modest positive correlation (ρ = 0.268, p < 0.001) with substantial scatter.* ***(C)*** *AGEs vs age: weak positive correlation (ρ = 0.140, p = 0.019).* ***(D)*** *AGEs vs total cholesterol (TC): weak negative correlation (ρ = -0.176, p = 0.003).* ***(E)*** *AGEs vs triglycerides (TG): no significant correlation (ρ = -0.001, p = 0.983).* ***(F)*** *AGEs vs blood urea nitrogen (BUN): weak positive correlation (ρ = 0.091, p = 0.127, non-significant). Collectively, these data demonstrate that AGEs correlate most strongly with glycemic control markers, particularly HbA1c, while showing minimal association with lipid parameters and renal function markers.*

## Figure S4. Progressive Glycemic Parameter Changes Across Disease Stages


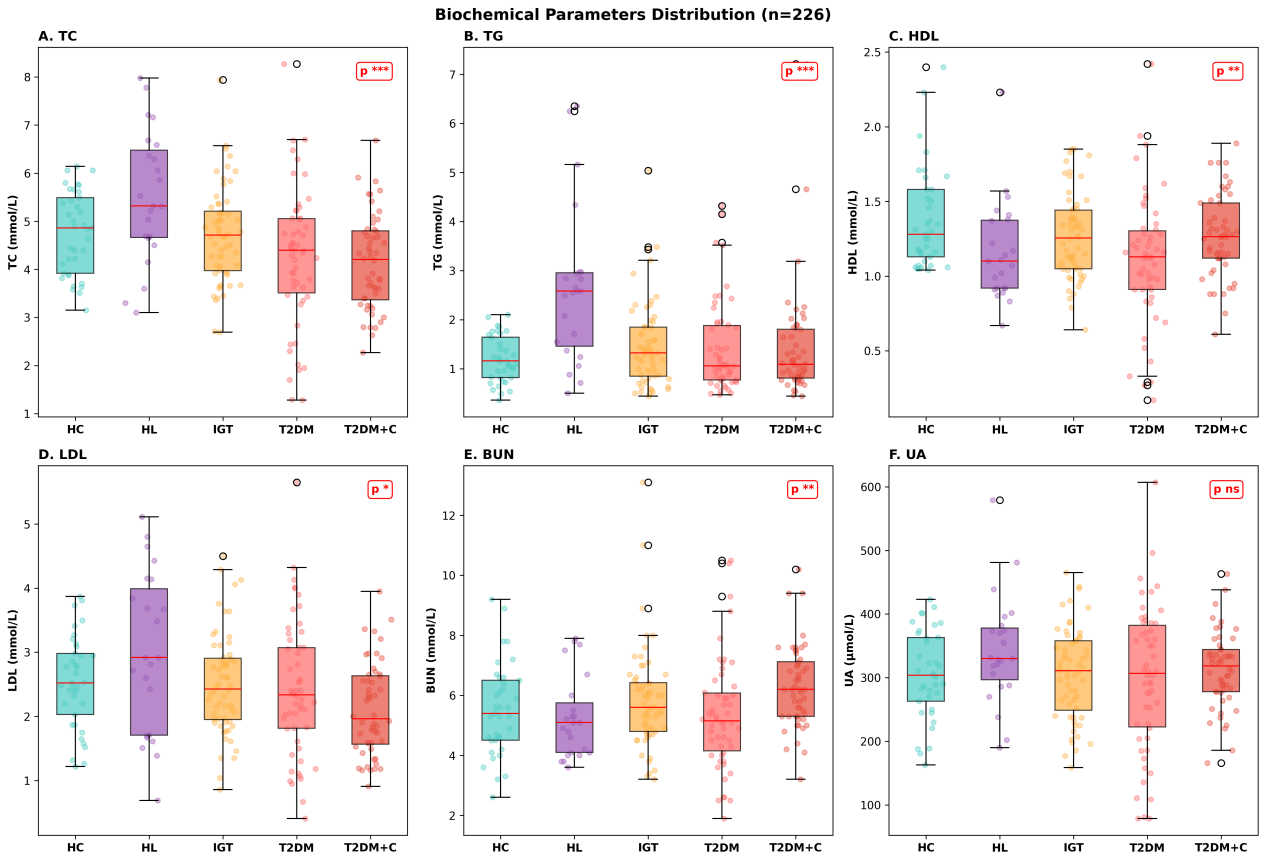


*Box plots with individual data points showing distributions of six biochemical parameters across five disease groups (HC, HL, IGT, T2DM, T2DM+C).* ***(A)*** *Total cholesterol (TC) and* ***(B)*** *triglycerides (TG) were significantly elevated in HL (p***), with progressive normalization toward diabetic stages.* ***(C)*** *HDL showed significant group differences (p**), with lowest levels in T2DM.* ***(D)*** *LDL was significantly elevated in HL (p*).* ***(E)*** *BUN demonstrated significant group variation (p**), with highest values in T2DM+C.* ***(F)*** *Uric acid showed no significant differences across groups (ns). Color coding: HC (teal), HL (purple), IGT (yellow), T2DM (pink), T2DM+C (red)*

## Figure S4. **Progressive Glycemic Parameter Changes Across Disease Stages**


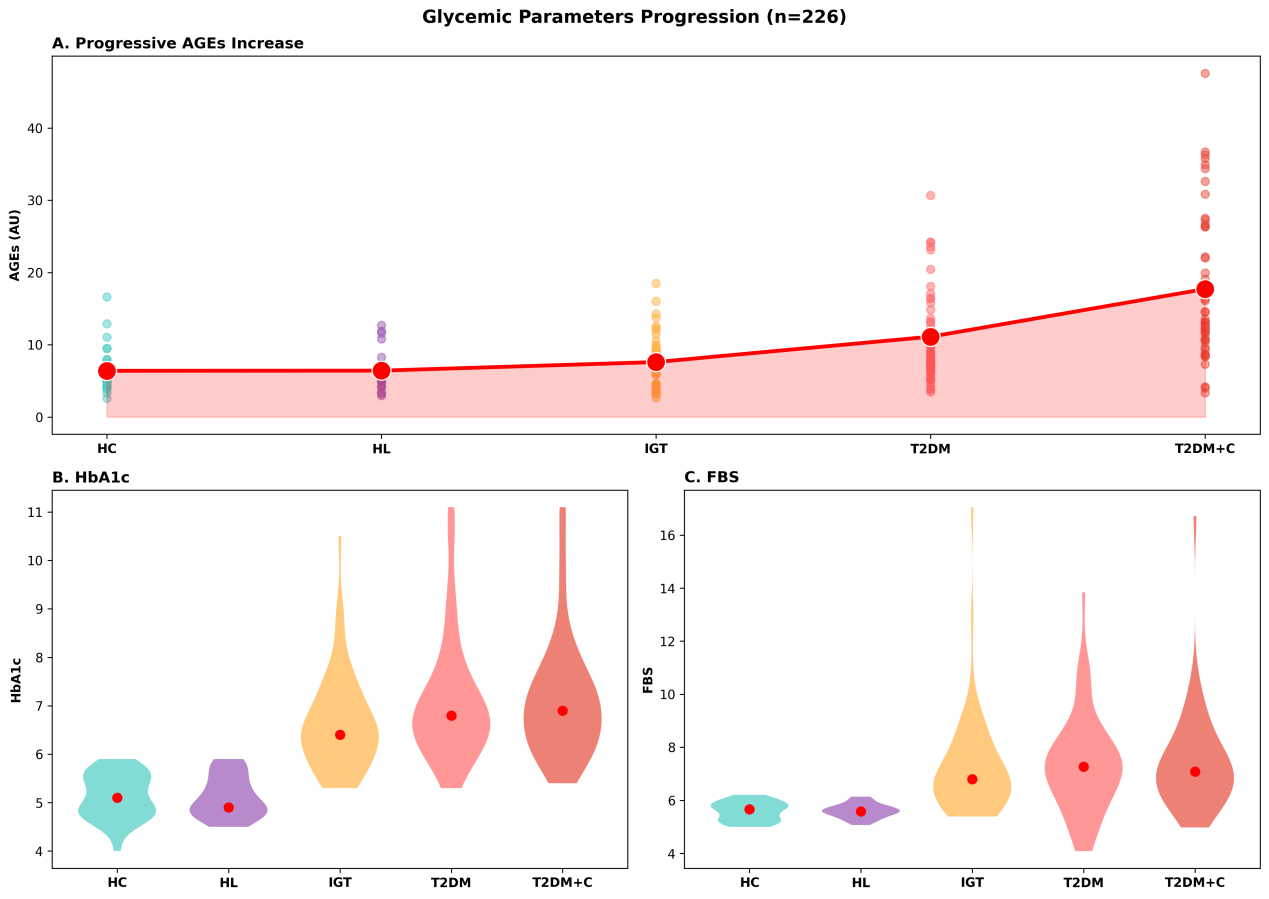


*(A) AGEs demonstrated exponential progression from HC/HL (median ~6.1 AU) through IGT (median ~7.2 AU) and T2DM (median ~9.8 AU) to T2DM+C (median ~13.4 AU), with marked variance expansion in advanced stages. (B-C) HbA1c and FBS showed stepwise elevations from normoglycemic ranges in HC/HL (~5% and ~5.5 mmol/L) to diabetic ranges in T2DM/T2DM+C (~7% and ~7 mmol/L). Red dots indicate group means; shaded area in (A) highlights the accelerating AGEs trajectory. Data are presented as median (interquartile range) with individual data points overlaid.*.

## Figure S5. Glycemic Correlations


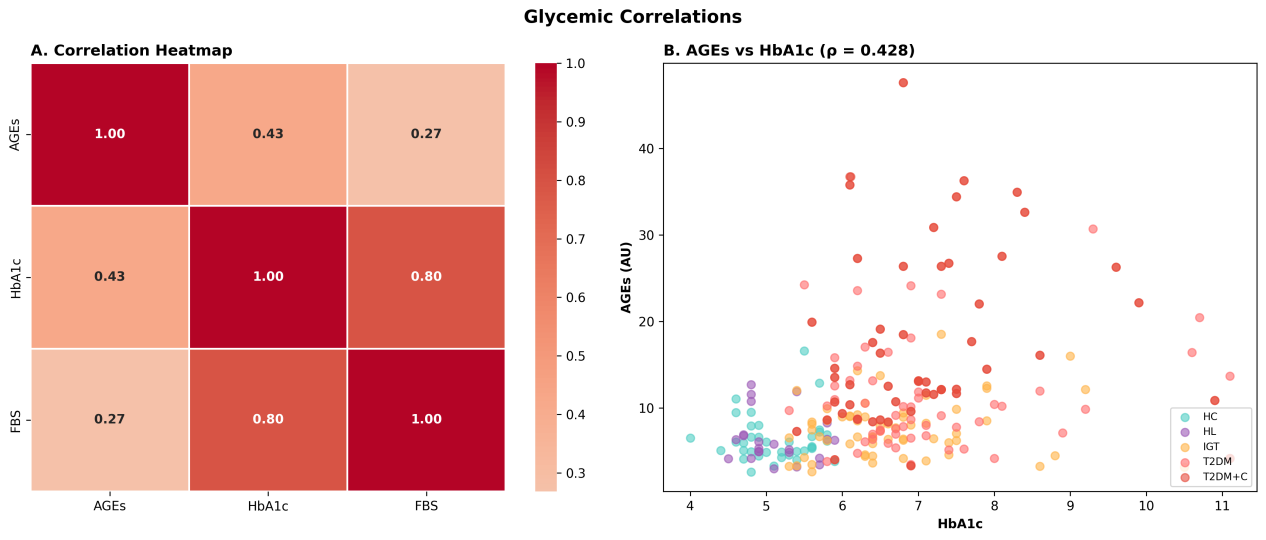


*Correlation heatmap and scatter plot showing relationships between AGEs and glycemic parameters. AGEs correlated moderately with HbA1c (ρ = 0.43) and modestly with FBS (ρ = 0.27), while HbA1c and FBS showed strong intercorrelation (ρ = 0.80)..Partial correlations controlling for age remained significant for AGEs-HbA1c (ρ = 0.396, p < 0.001) and AGEs-FBS (ρ = 0.219, p < 0.001).*

## Figure S6. Lipid Correlations


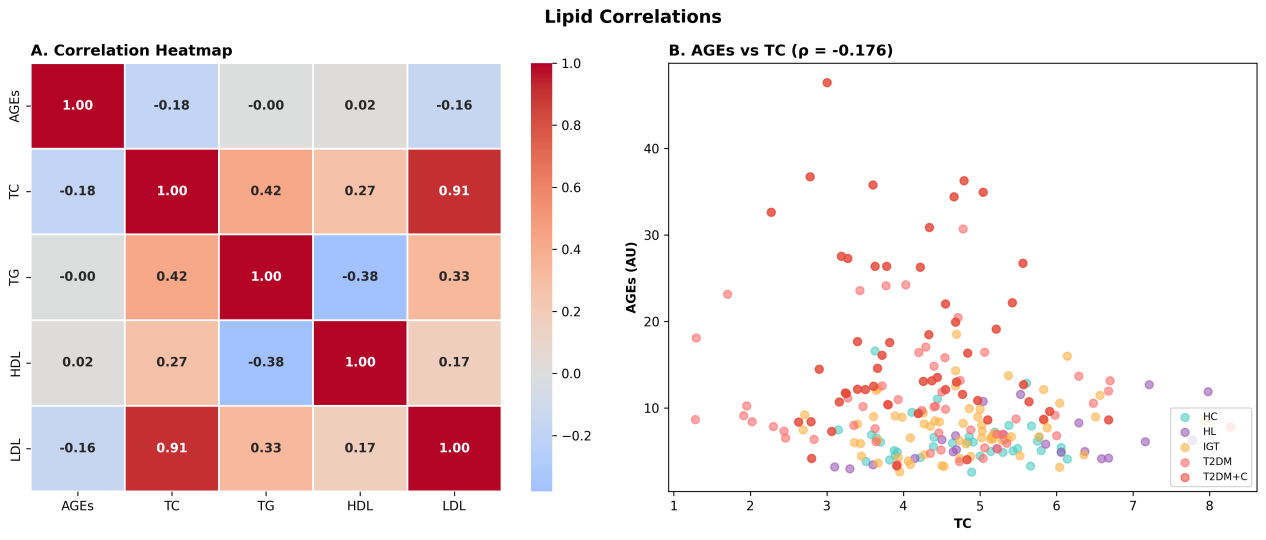


*Correlation matrix and scatter plot of AGEs with lipid parameters. AGEs showed weak negative correlation with total cholesterol (ρ = -0.18) and no correlation with triglycerides (ρ = -0.00). Strong positive correlations were observed between TC and LDL (ρ = 0.91) and TC and TG (ρ = 0.42).*

## Figure S7. Renal Correlations


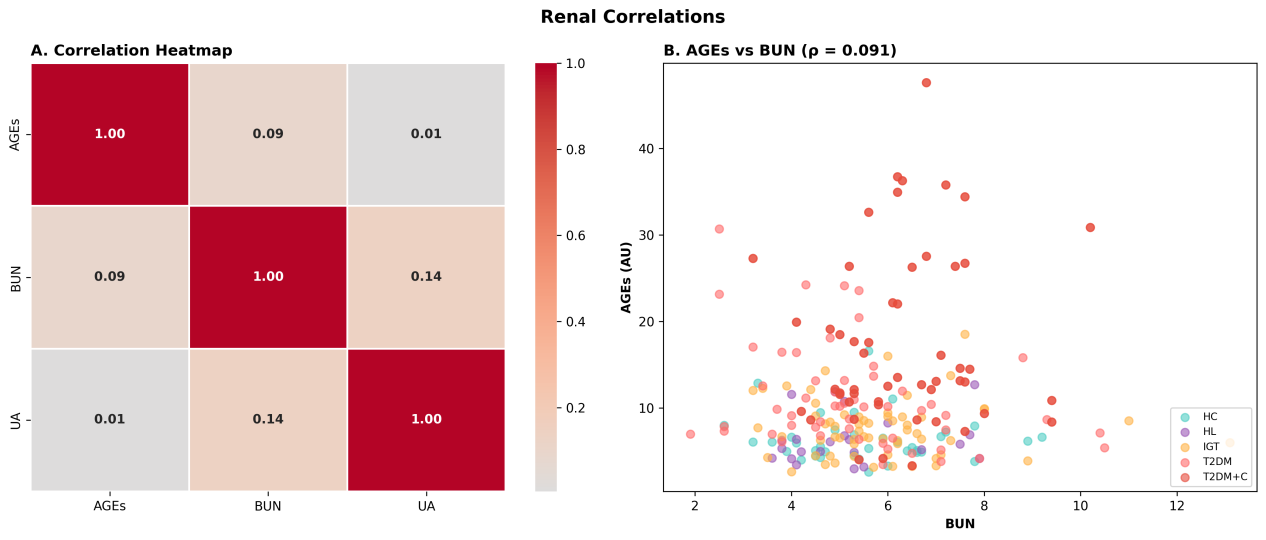


*Correlation heatmap and scatter plot of AGEs with renal function markers. AGEs demonstrated no meaningful correlation with BUN (ρ = 0.09) or uric acid (ρ = 0.01). BUN and UA showed weak positive correlation (ρ = 0.14).*

## Figure S8. Liver Correlations


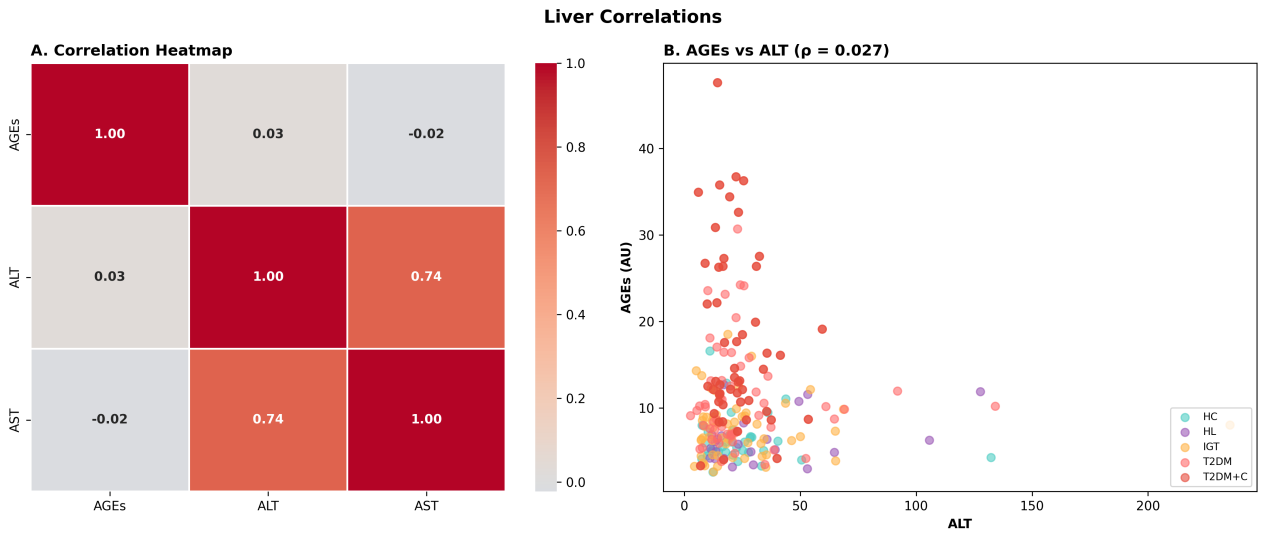


*Correlation matrix and scatter plot of AGEs with liver enzymes. AGEs showed no correlation with ALT (ρ = 0.03) or AST (ρ = -0.02). ALT and AST were strongly correlated (ρ = 0.74), consistent with their shared hepatic origin.*

## Figure S9. Comprehensive Correlations


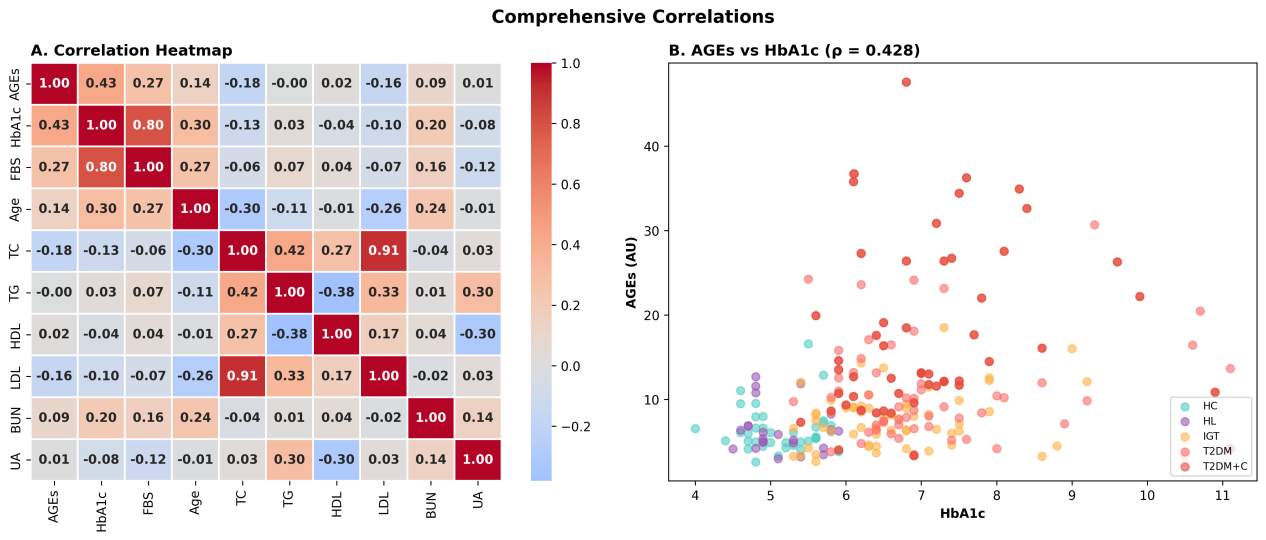


*Full correlation matrix of all 10 parameters with representative AGEs vs HbA1c scatter plot. Key findings: strongest correlations were HbA1c-FBS (ρ = 0.80), TC-LDL (ρ = 0.91), and ALT-AST (ρ = 0.74). AGEs showed strongest association with HbA1c (ρ = 0.43) among all parameters*

**Supplementary Tables**

## Table S1. Demographic and Clinical Characteristics

Baseline characteristics of 226 participants stratified by disease stage. Values are presented as median (interquartile range [IQR]) for continuous variables, with mean ± standard deviation provided parenthetically. Categorical variables are presented as n (%). HC = healthy controls; HL = hyperlipidemia; IGT = impaired glucose tolerance; T2DM = type 2 diabetes mellitus; T2DM+C = T2DM with complications; AGEs = advanced glycation end-products; AU = arbitrary units; HbA1c = glycated hemoglobin; FBS = fasting blood sugar.

| Group (n) | Age, years | Male, n (%) | AGEs, AU | HbA1c, % | FBS, mmol/L |
| --- | --- | --- | --- | --- | --- |
| HC (37) | 51.0 (46.0–57.0) *51.6 ± 8.9* | 13 (35.1%) | 5.99 (4.92–6.92) *6.40 ± 2.71* | 5.10 (4.80–5.60) *5.13 ± 0.46* | 5.67 (5.29–5.87) *5.59 ± 0.36* |
| HL (23) | 53.0 (47.0–58.0) *51.7 ± 9.1* | 13 (56.5%) | 5.86 (4.56–6.88) *6.42 ± 2.82* | 4.90 (4.80–5.40) *5.12 ± 0.43* | 5.58 (5.45–5.78) *5.60 ± 0.27* |
| IGT (60) | 59.0 (51.0–63.2) *57.5 ± 8.8* | 31 (51.7%) | 7.22 (5.58–9.01) *7.61 ± 3.27* | 6.40 (6.07–7.10) *6.64 ± 0.99* | 6.80 (6.04–7.70) *7.22 ± 1.88* |
| T2DM (54) | 61.5 (53.2–70.0) *60.3 ± 10.6* | 29 (53.7%) | 9.81 (7.04–13.20) *11.12 ± 5.95* | 6.80 (6.30–7.38) *7.09 ± 1.25* | 7.27 (6.16–8.13) *7.34 ± 1.84* |
| T2DM+C (52) | 61.0 (55.8–67.0) *60.1 ± 8.1* | 37 (71.2%) | 13.35 (10.73–26.32) *17.72 ± 10.13* | 6.90 (6.20–7.50) *7.11 ± 1.22* | 7.09 (6.16–7.99) *7.39 ± 1.85* |

## Table S2. Biochemical Parameters Across Disease Stages

Lipid profile and renal function markers across five disease groups. Values are median (interquartile range [IQR]), with mean ± standard deviation in italics. TC = total cholesterol; TG = triglycerides; HDL = high-density lipoprotein; LDL = low-density lipoprotein; BUN = blood urea nitrogen; UA = uric acid. All values in mmol/L except UA (μmol/L). Non-parametric Kruskal-Wallis tests were used for between-group comparisons.

| Group (n) | TC (mmol/L) | TG (mmol/L) | HDL (mmol/L) | LDL (mmol/L) | BUN (mmol/L) | UA (μmol/L) |
| --- | --- | --- | --- | --- | --- | --- |
| HC (37) | 4.86 (3.92–5.49) *4.76 ± 0.86* | 1.16 (0.82–1.64) *1.19 ± 0.47* | 1.28 (1.13–1.58) *1.38 ± 0.34* | 2.52 (2.03–2.98) *2.54 ± 0.72* | 5.40 (4.50–6.50) *5.52 ± 1.53* | 304 (263–363) *305 ± 70* |
| HL (23) | 5.32 (4.67–6.48) *5.52 ± 1.36* | 2.58 (1.46–2.96) *2.64 ± 1.60* | 1.10 (0.92–1.38) *1.16 ± 0.34* | 2.92 (1.71–3.99) *2.98 ± 1.26* | 5.10 (4.10–5.75) *5.26 ± 1.38* | 330 (297–378) *343 ± 86* |
| IGT (60) | 4.71 (3.97–5.21) *4.71 ± 1.00* | 1.33 (0.85–1.85) *1.48 ± 0.87* | 1.25 (1.05–1.44) *1.27 ± 0.29* | 2.42 (1.95–2.90) *2.50 ± 0.78* | 5.60 (4.80–6.43) *5.84 ± 1.69* | 311 (249–358) *306 ± 72* |
| T2DM (54) | 4.40 (3.51–5.05) *4.24 ± 1.44* | 1.06 (0.77–1.88) *1.41 ± 0.92* | 1.13 (0.91–1.30) *1.11 ± 0.43* | 2.33 (1.81–3.07) *2.42 ± 1.06* | 5.15 (4.15–6.07) *5.33 ± 1.85* | 307 (223–382) *298 ± 114* |
| T2DM+C (52) | 4.21 (3.37–4.80) *4.15 ± 0.99* | 1.09 (0.81–1.81) *1.41 ± 1.11* | 1.27 (1.12–1.49) *1.27 ± 0.28* | 1.96 (1.57–2.63) *2.11 ± 0.72* | 6.20 (5.30–7.12) *6.30 ± 1.37* | 319 (278–344) *313 ± 60* |

## Table S3. Pairwise Comparisons of Key Variables

Post-hoc pairwise comparisons with effect sizes for AGEs, FBS, and HbA1c across disease stages. P-values derived from Dunn's test with Bonferroni correction for 10 comparisons. Effect sizes interpreted as: small (d = 0.2–0.5), medium (d = 0.5–0.8), large (d > 0.8). Note: The HC vs IGT comparison for AGEs did not remain significant after Bonferroni correction (p = 0.094), despite a small-to-medium effect size (d = 0.425).

| Variable | Comparison | p-value (Dunn's + Bonferroni) | Uncorrected p | Cohen's d | Effect Size |
| --- | --- | --- | --- | --- | --- |
| AGEs (AU) | HC vs IGT | **0.094** | 0.037 | 0.425 | Small (trend) |
| AGEs (AU) | HC vs T2DM | <0.001 | <0.001 | 1.027 | Large |
| AGEs (AU) | HC vs T2DM+C | <0.001 | <0.001 | 1.540 | Large |
| AGEs (AU) | IGT vs T2DM | 0.013 | 0.001 | 0.721 | Medium |
| AGEs (AU) | IGT vs T2DM+C | <0.001 | <0.001 | 1.346 | Large |
| AGEs (AU) | T2DM vs T2DM+C | 0.013 | 0.001 | 0.802 | Large |

## Table S4. AGEs Levels by Complication Burden in T2DM+C Patients

Stratification of 52 T2DM+C patients by number of complications. Values are mean ± standard deviation (median and interquartile range in italics). Kruskal-Wallis test: H = 5.64, p = 0.060 for AGEs across complication count groups.

| Complication Count | n | AGEs (AU) | HbA1c (%) / FBS (mmol/L) |
| --- | --- | --- | --- |
| 1 Complication | 31 | 15.02 ± 8.80 Median 13.2 (IQR 9.8–18.5) | HbA1c: 7.18 ± 1.41 FBS: 7.54 ± 2.11 |
| 2 Complications | 16 | 21.77 ± 11.86 Median 19.4 (IQR 12.8–28.1) | HbA1c: 6.95 ± 0.95 FBS: 7.21 ± 1.51 |
| ≥3 Complications | 5 | 21.53 ± 7.69 Median 21.9 (IQR 16.3–25.7) | HbA1c: 7.22 ± 0.72 FBS: 7.00 ± 1.07 |

## Table S5. ROC Analysis for Clinical Staging

Diagnostic performance of AGEs for distinguishing adjacent disease stages. Area under the curve (AUC) values include 95% confidence intervals derived from 2,000 bootstrap resamples. Optimal thresholds determined by Youden's index. Sensitivity and specificity reported at optimal cutoff. These thresholds are exploratory and require external validation.

| Comparison | AUC (95% CI) | Optimal Threshold (AU) | Sensitivity | Specificity | Clinical Interpretation |
| --- | --- | --- | --- | --- | --- |
| HC → IGT | 0.627 (0.512–0.736) | 6.265 | 0.700 | 0.622 | Limited screening utility; modest discrimination |
| IGT → T2DM | 0.691 (0.589–0.783) | 9.134 | 0.556 | 0.783 | Moderate staging utility |
| T2DM → T2DM+C | 0.723 (0.621–0.813) | 10.719 | 0.769 | 0.630 | Best performance for complication detection |

## Table S6. ROC Analysis for Biomarker Validation

Comprehensive diagnostic performance of AGEs for discriminating healthy controls from individual disease groups and all disease groups combined. AUC values include 95% confidence intervals from 2,000 bootstrap resamples. Sample sizes for each comparison provided. Optimal thresholds are exploratory.

| Comparison | AUC (95% CI) | Optimal Threshold | Sensitivity | Specificity | n (Group 1) | n (Group 2) |
| --- | --- | --- | --- | --- | --- | --- |
| HC vs HL | 0.486 (0.331–0.643) | 10.798 | 0.174 | 0.919 | 37 | 23 |
| HC vs IGT | 0.627 (0.512–0.736) | 6.265 | 0.700 | 0.622 | 37 | 60 |
| HC vs T2DM | 0.799 (0.705–0.888) | 6.993 | 0.778 | 0.757 | 37 | 54 |
| HC vs T2DM+C | 0.908 (0.834–0.970) | 8.394 | 0.923 | 0.865 | 37 | 52 |
| HC vs All Disease | 0.736 (0.659–0.809) | 8.014 | 0.598 | 0.865 | 37 | 189 |

## Table S7. Multivariable Regression Analyses

Table S7a. Multivariable Logistic Regression: T2DM+C vs Healthy Controls. Model adjusted for age, sex, and HbA1c. AGEs remained an independent predictor of complicated diabetes after comprehensive adjustment. 10-fold cross-validated AUC = 0.971 ± 0.024.

| Predictor | Coefficient (β) | Odds Ratio (OR) | 95% CI for OR |
| --- | --- | --- | --- |
| AGEs (per AU) | 1.594 | 4.92 | [2.98–8.98] |
| HbA1c (per %) | 2.684 | 14.65 | [9.64–21.21] |
| Age (per year) | 0.664 | 1.94 | [1.21–3.16] |
| Male sex | 0.640 | 1.90 | [1.21–2.99] |

Table S7b. Multiple Linear Regression: Predictors of Serum AGE Concentration (n = 226). R² = 0.168. HbA1c was the strongest independent predictor of serum AGEs. FBS showed a negative coefficient, likely reflecting multicollinearity with HbA1c (ρ = 0.80).

| Predictor | Standardized β | Interpretation |
| --- | --- | --- |
| HbA1c (%) | 2.756 | Strongest positive predictor |
| Male sex | 2.015 | Higher AGEs in males |
| LDL (mmol/L) | -1.554 | Inverse association |
| FBS (mmol/L) | -0.804 | Negative (multicollinearity with HbA1c) |
| TG (mmol/L) | -0.417 | Weak negative |
| TC (mmol/L) | 0.358 | Weak positive |
| BUN (mmol/L) | -0.140 | Non-significant |
| UA (μmol/L) | -0.001 | Non-significant |
